# Effects of different ovulation induction protocols on pregnancy outcomes of fresh cycles in patients undergoing in vitro fertilization-embryo transfer with donor sperm

**DOI:** 10.1007/s00404-025-08272-4

**Published:** 2026-01-08

**Authors:** Lixiao Su, Xiangyang Jing, Lin Zeng, Li Luo, Haiyan Wang, Rong Li, Hongbin Chi

**Affiliations:** 1 Reproductive Medicine Center, Peking University Third Hospital, No. 49, North Garden Road, Haidian District, Beijing, 100191 China; 2 Reproductive Medicine Center, Women and Infants Hospital of Zhengzhou, No. 41, Jinshui Road, Jinshui District, Zhengzhou, 450000 China; 3 Obstetrics Department, Affiliated Hospital of Chengde Medical University, No. 36, Nanyingzi Street, Shuangqiao District, Chengde, 067000 China; 4 Clinical Epidemiology Research Center, Peking University Third Hospital, No.49, North Garden Road, Haidian District, Beijing, 100191 China

**Keywords:** Donor sperm, In vitro fertilization-embryo transfer, Ovulation induction protocols, Fresh cycles, Pregnancy outcomes

## Abstract

**Objectives:**

This study analyzed clinical pregnancy outcomes in patients undergoing in vitro fertilization-embryo transfer with donor sperm (IVF-D) using different ovulation induction protocols, to provide reference data for selecting appropriate protocols.

**Methods:**

Data from 1801 cycles in patients who underwent IVF-D in Peking University Third Hospital between June 2010 and June 2021 were retrospectively analyzed. Participants were divided into three groups based on the controlled ovarian hyperstimulation protocol: follicular-phase ultralong gonadotropin-releasing hormone agonist (GnRH-a), luteal-phase GnRH-a long, and gonadotropin-releasing hormone antagonist (GnRH-ant) protocol groups.

**Results:**

Significant differences were observed among the groups in gonadotropin (Gn) starting dose, Gn administration duration, total Gn dose, estradiol level on the day of human chorionic gonadotropin (hCG) administration (hCG day), progesterone level on hCG day, luteinizing hormone level on hCG day, endometrial thickness on hCG day, and number of embryos transferred (p < 0.05). We also found significant group differences in the number of eggs retrieved, two pronucleizygotes, and cleavages (p < 0.05), but not in high-quality embryos (p < 0.05). Clinical pregnancy and live birth rates significantly differed among the three groups (p < 0.05), whereas ectopic pregnancy, early miscarriage, and multiple pregnancy rates did not (p < 0.05).

**Conclusion:**

In fresh embryo transfer cycles, the GnRH-ant protocol required the shortest duration of Gn administration and lowest total Gn dose, whereas the GnRH-a long protocol had the highest clinical pregnancy rate. Therefore, the GnRH-a long protocol is considered the preferred method for female patients who can undergo fresh transfers during IVF-D cycles.

## What does this study adds to the clinical work

**Table:** 

Infertility affects approximately 15% of the global population, with male factors contributing to a significant portion of cases. This study compares different ovulation induction protocols in IVF using donor sperm, providing valuable insights into their impact on clinical pregnancy outcomes.	

## Introduction

The incidence of infertility is increasing annually, affecting approximately 15% of the population of childbearing age. The “Infertility Prevalence Estimates (1990–2021)” report released by the World Health Organization [1] estimates that approximately 17.5% of adults (one-sixth of the population) worldwide are affected by infertility, with an increasing trend. Infertility has become a major public health problem. Approximately 30% of infertility cases are attributed to male factors. Among male infertility cases, azoospermia accounts for an estimated 10–15%, with various underlying causes, although the mechanisms remain unclear [2]. For men affected by irreversible azoospermia, assisted reproductive technologies such as artificial insemination by donor (AID) and in vitro fertilization with donor sperm (IVF-D) or intracytoplasmic sperm injection with donor sperm can be adopted, depending on the female fertility status. Currently, the follicular-phase ultralong gonadotropin-releasing hormone agonist (GnRH-a) protocol, luteal-phase GnRH-a long protocol, and gonadotropin-releasing hormone antagonist (GnRH-ant) protocol are the main controlled ovarian hyperstimulation approaches used in assisted reproductive technologies. The ultralong GnRH-a protocol and GnRH-a long protocol may help prevent the premature elevation of luteinizing hormone (LH) levels, increase the number of eggs retrieved and pregnancy rates, and reduce the number of cycle cancelations [3, 4]. In contrast, GnRH-ant competitively blocks GnRH receptors and rapidly inhibits the release of gonadotropin (Gn) [5], reducing the risk of ovarian hyperstimulation syndrome.

This study aimed to provide a reference for the selection of the appropriate ovulation induction protocol in patients undergoing IVF-D by retrospectively analyzing the clinical pregnancy outcomes of patients undergoing IVF-D using the follicular-phase ultralong GnRH-a protocol, luteal-phase GnRH-a long protocol, or GnRH-ant protocol.

## Methods

### Study participants

Data from 1801 cycles in patients who underwent IVF-D in the Peking University Third Hospital between June 2010 and June 2021 were collected for retrospective analysis. The study population was divided into three groups according to the adopted controlled ovarian hyperstimulation protocols.

1. Group treated with the follicular-phase ultralong GnRH-a protocol. Pregnancy was excluded on the first day of menstruation and administer 3.75 mg of GnRH agonist, and serum follicle-stimulating hormone (FSH), LH, estradiol (E2), and progesterone (P) level testing and B-mode ultrasound monitoring were performed on days 28–35. Ovulation was induced with Gn after full pituitary downregulation was achieved (endometrial thickness ≤ 5 mm, blood LH < 5 mIU/mL, blood FSH < 5 mIU/mL, blood E2 < 200 pmol/L, blood P < 3.68 nmol/mL, absence of follicles with a diameter ≥ 10 mm, or functional cysts in both ovaries).
2. Group treated with the GnRH-a long protocol (i.e., luteal-phase short- or long-acting GnRH-a long protocol). Administration of the short-acting GnRH-a was initiated 7–10 days before menstruation (mid-luteal phase), whereas the long-acting GnRH-a was administered in the middle-to-late luteal phase. Pregnancy was excluded after 10–14 days of downregulation or on the second day of menstruation, and serum FSH, LH, E2 levels, and P testing and B-mode ultrasound were performed. Gn drugs were administered after 14–21 days of downregulation when the target criterion was reached.
3. Group treated with the GnRH-ant protocol. Serum FSH, LH, E2, and P testing, B-mode ultrasound monitoring, and urine pregnancy test were performed on the second day of menstruation. If cysts and large follicles (< 9 mm) were not detected by ultrasound, the urine pregnancy test was negative, and serum hormones were at the basal levels, Gn injection was initiated on days 2–3 of menstruation. The antagonist was added on day 6 of Gn injection or when the follicular diameter was > 12 mm.

**Fig. 1:**
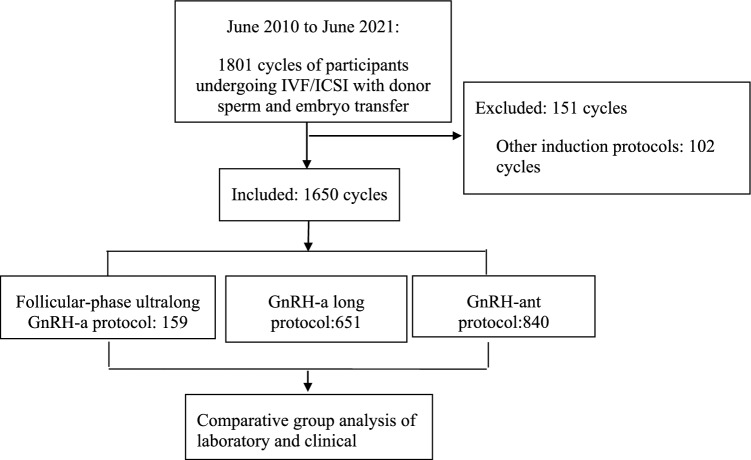
Flowchart of the screening process of participant enrolment. GnRH-a, gonadotropin-releasing hormone agonist; GnRH-ant, gonadotropin-releasing hormone antagonist; ICSI, intracytoplasmic sperm injection with donor sperm; IVF, in vitro fertilization.

A flowchart detailing the study process is presented in Fig. 1.

### Study eligibility criteria

The participants were included if they met the following criteria:

1. Male participant diagnosed with azoospermia.
2. The female partner of this participant failed to get pregnant by AID (blood chorionic gonadotropin was < 5 mIU/mL at 16 days after AID) at least three times.
3. Azoospermia in combination with fallopian tube obstruction, polycystic ovary syndrome, endometriosis, or ovarian insufficiency.
4. Receiving IVF-D for the first time, and fresh transfer cycles were used.

The participants were excluded if they met the following criteria:

1. Abnormal development of the uterus in the female participant, such as unicornuate uterus, septate uterus, and double uterus.
2. Other uterine factors in the female participant, such as endometrial polyps, leiomyomas, and adenomyosis.
3. Recurrent abortions.
4. At least three failed attempts in previous IVF-Ds.

### Trigger timing

If two follicles with diameters of ≥ 18 mm were detected, it was checked whether the serum LH, E2, and P levels were consistent with the number of follicles. If they were consistent, human chorionic gonadotropin (hCG; genetically recombinant hCG 250 µg or urogenic hCG 5,000–10,000 IU) was administered to promote final maturation of primary oocytes prior to ovulation. Eggs were retrieved 36–38 h after hCG injection.

### Embryo transfer and luteal-phase support

According to the “Specifications for Assisted Reproductive Technology Operations” issued by the Chinese Ministry of Health, 1–2 cleavage-stage embryos were transferred on day 3 post-egg retrieval, or a single blastocyst was transferred on day 5. The embryo transfer was conducted by designated reproductive physicians adhering to standardized criteria. After fresh transfer, oral dydrogesterone 20 mg/day combined with vaginal progesterone vaginal gel 90 mg/day or progesterone soft capsules 400 mg/day was given for luteal-phase support until 8–10 weeks of intrauterine pregnancy.

### Donor sperm preparation

Donor sperm samples were provided by the National Human Sperm Bank. The general procedures for donor recruitment, sperm freezing, and receiver selection complied with the National Health and Family Planning Commission of the People’s Republic of China standards. All donor samples were regularly evaluated to eliminate the risk of sexually transmitted and genetic diseases. After selecting the donor sample that matched the phenotypic characteristics and blood types of the receiving couple, the sample was thawed and prepared by density gradient technique when needed.

After thawing, sperm were treated using the upstream method. ICSI was used for sperm concentrations below 15 million, and IVF was used for pregnancy assistance.

### Determination of laboratory outcomes

High-quality embryos were defined as embryos derived from normal fertilized eggs with ≥ 5 embryonic cells on the third day after fertilization, the cell size in line with the developmental stage, a fragmentation degree of < 10%, and without multinucleated cells.

### Determination of pregnancy outcomes

Clinical pregnancy was defined as the detection of one or more gestational sacs on ultrasound, including normal intrauterine pregnancy, ectopic pregnancy, and combined intrauterine and extrauterine pregnancy, including cases where only a gestational sac without the fetal heart was observed [6]. The live birth rate referred to the number of births resulting in at least one live birth per embryo transfer cycle. After confirmation of pregnancy, any spontaneous abortion within 12 weeks of gestation (excluding biochemical pregnancies) was defined as early miscarriage. Ectopic pregnancy referred to the implantation of the gestational sac outside the uterine cavity, including extrauterine pregnancy and combined intrauterine and extrauterine pregnancy. A multiple pregnancy was defined as a pregnancy in which two or more fetuses were conceived at the same time.

The following formulas were used for calculations:

Clinical pregnancy rate = number of clinical pregnancies / number of transfer cycles × 100%

Live birth rate = number of live births / number of transfer cycles × 100%

Early miscarriage rate = number of early miscarriages / number of clinical pregnancies × 100%

Ectopic pregnancy rate = number of ectopic pregnancies / number of clinical pregnancies × 100%

Multiple pregnancy rate = number of multiple pregnancies / number of clinical pregnancies × 100%.

### Statistical analysis

SPSS 25.0 (IBM SPSS Statistics 25.0, Internation Business Machines Corp.) software was used for all analyses. The normality of data distribution was tested before using the ANOVA for continuous variable analysis. The Chi-square test was used for categorical data analysis. Data with normal or approximately normal distribution were expressed as mean ± standard deviation, and p values of < 0.05 were considered statistically significant.

Binary logistic regression analysis was performed to calculate adjusted relative risk estimates of clinical pregnancy and miscarriage rates, adjusting for factors, such as age, body mass index, endometrial thickness, number of embryos transferred, number of eggs retrieved, and number of high-quality embryos. Odds ratio (OR) and 95% confidence intervals (CI) were estimated for each exposed cohort relative to the unexposed cohort.

### Ethical review

This study was approved by the Ethics Committee of Peking University Third Hospital (approval number M2023577). The authors confirm that all experiments were performed in accordance with the principles of the Declaration of Helsinki.

**Results**

### Comparison of the baseline characteristics among the three study groups

We found no significant differences in baseline infertility type, duration of infertility, female age, body mass index, and baseline E2, FSH, and LH levels among the three study groups (Table 1).

**Table 1:** Comparison of the baseline data among the three study groups

	Follicular-phase ultralong GnRH-a protocol	GnRH-a long protocol	GnRH-ant protocol	p value
	n = 159	n = 651	n = 840	
*Types of infertility* (n/%)				
Primary	138 (86.79)	511 (78.49)	677 (80.60)	0.06
Secondary	21 (13.21)	140 (21.51)	163 (19.40)	
Duration of infertility (years)	5.23 ± 3.63	4.94 ± 3.80	5.07 ± 3.53	0.64
*Source of infertility* (n/%)				0.00
Combined with a female factor	66 (41.51)^b^	184 (28.26)^ac^	318 (37.86)^b^	
Combined with Diminished Ovarian Reserve (DOR)	10	39	84	
Combined with Polycystic Ovary Syndrome(PCOS)	9	20	43	
Combined with Endometriosis	21	23	38	
Combined with Pelvic and Tubal Factors		106	180	
Not combined with a female factor	93 (58.49)^b^	467 (71.74)^ac^	522 (62.14)^b^	
Female Age (years)	31.44 ± 4.48	31.29 ± 4.30	31.45 ± 4.45	0.78
Body mass index (kg/m^2^)	22.79 ± 3.40	22.59 ± 3.35	22.64 ± 3.29	0.81
Baseline E2 level (pmol/L)	170.77 ± 70.28	166.72 ± 75.21	167.80 ± 79.30	0.84
Baseline FSH level (mIU/mL)	6.85 ± 2.89	6.96 ± 2.91	6.88 ± 2.14	0.79
Baseline LH level (mIU/mL)	4.09 ± 2.98	3.96 ± 2.07	4.12 ± 2.42	0.41
*Fertilization method* (n/%)				
ICSI	130 (81.76)^c^	491 (75.42)^c^	574 (68.33)^ab^	0.00
IVF	29 (18.24)^c^	160 (24.58)^c^	266 (31.67)^ab^	
Cleavage-stage embryos (n/%)	147 (92.45)	607 (93.24)	786 (93.57)	0.87
Blastocyst (n/%)	12 (7.55)	44 (6.76)	54 (6.43)	

E2, estradiol; FSH, follicle-stimulating hormone; GnRH-a, gonadotropin-releasing hormone agonist; GnRH-ant, gonadotropin-releasing hormone antagonist; ICSI, intracytoplasmic sperm injection with donor sperm; IVF, in vitro fertilization; LH, luteinizing hormone

^A^Compared with follicular-phase ultralong GnRH-a protocol

^b^Compared with GnRH-a long protocol

^c^Compared with GnRH-ant protocol

In pairwise comparisons, infertility factors and fertilization modes showed statistical differences among the GnRH-ant protocol group, the follicular-phase ultralong GnRH-a protocol, and the GnRH-a long protocol group (Table 2).

**Table 2:** Comparison of the ovulation induction cycle data among the three study groups

	Follicular-phase ultralong GnRH-a protocol	GnRH-a long protocol	GnRH-ant protocol	F value	p value
	n = 159	n = 651	n = 840		
E2 level on hCG day (pmol/L)	6258.70 ± 3446.39^bc^	7594.91 ± 3987.49^a^	7377.76 ± 3479.63^a^	8.430	0.00
P level on hCG day (nmol/mL)	2.25 ± 1.32	2.09 ± 1.09	2.23 ± 1.09	3.000	0.05
LH level on hCG day (mIU/mL)	0.63 ± 0.69^c^	0.77 ± 0.59^c^	2.42 ± 2.71^ab^	148.180	0.00
Gn starting dose (IU)	247.88 ± 99.98^c^	239.32 ± 89.58^c^	226.06 ± 82.51^ab^	6.680	0.00
Duration of Gn administration (days)	12.62 ± 2.03^c^	13.03 ± 2.07^c^	10.28 ± 1.57^ab^	444.029	0.00
Total Gn dose (IU)	3330.27 ± 1302.26^c^	3374.85 ± 1234.28^c^	2466.61 ± 924.55^ab^	139.192	0.00
Endometrial thickness on hCG day (mm)	11.53 ± 1.52^c^	11.34 ± 1.67^c^	10.81 ± 1.58^ab^	26.036	0.00
Number of embryos transferred (n)	1.91 ± 0.40	1.90 ± 0.37^c^	1.84 ± 0.38^b^	6.125	0.00

E2, estradiol; Gn, gonadotropin; GnRH-a, gonadotropin-releasing hormone agonist; GnRH-ant, gonadotropin-releasing hormone antagonist; hCG, human chorionic gonadotropin; LH, luteinizing hormone; P, progesterone

^a^Compared with follicular-phase ultralong GnRH-a protocol

^b^Compared with GnRH-a long protocol

^c^Compared with GnRH-ant protocol

### Comparison of the ovulation induction cycles

The three study groups significantly differed in the Gn starting dose, duration of Gn administration, total Gn dose, E2 level on the day of hCG administration (hCG day), LH level on hCG day, endometrial thickness on hCG day, and the number of embryos transferred (Table 2).

In pairwise comparisons, the number of days of Gn administration differed among the groups. This number was lowest in the GnRH-ant protocol groups, respectively. The GnRH-ant protocol group had the lowest Gn starting dose which was significantly different from the doses of the follicular-phase ultralong GnRH-a protocol and GnRH-a long protocol groups (Table 2). Likewise, the total Gn dose was significantly lower in the GnRH-ant protocol group than in the follicular-phase ultralong GnRH-a protocol and GnRH-a long protocol groups (Table 2). The E2 level on the hCG day showed a statistical difference among the three groups, with the follicular-phase ultralong GnRH-a protocol having the lowest E2 level (Table 2). The GnRH-ant protocol group had the highest LH level on hCG day (Table 2). Among the three groups, the GnRH-ant protocol group also had the thinnest endometrial thickness on hCG day (Table 2), with a statistical difference observed between the GnRH-ant protocol group and the GnRH-a long protocol group (Table 2).

### Comparison of laboratory outcomes

Significant group differences were found in the number of eggs retrieved, the number of zygotes with two pronuclei, and the number of cleavages, whereas the number of high-quality embryos was not significantly different (Table 3). Pairwise comparisons among the three study groups showed that the number of eggs retrieved was significantly different. The GnRH-ant protocol group had the least number of eggs retrieved in comparison to the follicular-phase ultralong GnRH-a protocol and GnRH-a long protocol groups. Additionally, the number of zygotes with two pronuclei and the number of cleavages were lowest in the GnRH-ant protocol group (Table 3).

**Table 3:** Comparison of the laboratory outcomes among the three study groups

	Follicular-phase ultralong GnRH-a protocol	GnRH-a long protocol	GnRH-ant protocol	F value	p value
	n = 159	n = 651	n = 840		
Number of eggs retrieved (n)	11.18 ± 4.39^c^	10.97 ± 4.63^c^	10.22 ± 4.59^ab^	6.282	0.00
Number of 2PN zygotes (n)	6.39 ± 3.29	6.43 ± 3.26^c^	5.98 ± 3.31^b^	3.826	0.02
Fertilization rate of zygotes (%)	57.68	59.92	59.51	0.742	0.48
Number of cleavages (n)	7.31 ± 3.38	7.31 ± 3.49^c^	6.76 ± 3.57^b^	4.911	0.00
Number of high-quality embryos (n)	4.52 ± 2.75	4.53 ± 2.88	4.27 ± 2.76	1.728	0.18

2PN, two pronuclei; GnRH-a, gonadotropin-releasing hormone agonist; GnRH-ant, gonadotropin-releasing hormone antagonist

^a^Compared with follicular-phase ultralong GnRH-a protocol

^b^Compared with GnRH-a long protocol; c: compared with GnRH-ant protocol

### Comparison of clinical outcomes

The clinical pregnancy and live birth rates were significantly different among the three study groups, whereas no significant differences in ectopic pregnancy, early miscarriage, and multiple pregnancy rates were observed (Table 4). The clinical pregnancy rate was in pairwise comparisons significantly lower in the GnRH-ant protocol group than in the GnRH-a long protocol group (Table 4). In pairwise comparisons, the live birth rate was significantly lower in the GnRH-ant protocol group than in the GnRH-a long protocol group (Table 4).

**Table 4:** Comparison of the clinical outcomes among the three groups

	Follicular-phase ultralong GnRH-a protocol	GnRH-a long protocol	GnRH-ant protocol	X^2^ value	p value
	n = 159	n = 651	n = 840		
Clinical pregnancy rate (%)	52.8	56.8^c^	46.4^b^	16.096	0.00
Live birth rate (%)	45.3	49^c^	38.8^b^	20.486	0.00
Early miscarriage rate (%)	11.9	12.7	13.6	0.237	0.89
Ectopic pregnancy rate (%)	1.2	0.8	2.8	4.577	0.10
Multiple pregnancy rate (%)	34.5	34.3	31.6	11.069	0.09

GnRH-a, gonadotropin-releasing hormone agonist; GnRH-ant, gonadotropin-releasing hormone antagonist

^b^Compared with GnRH-a long protocol

^c^Compared with GnRH-ant protocol

### Binary logistic regression analysis of factors affecting the clinical pregnancy rate

After adjusting for factors such as age, body mass index, endometrial thickness on hCG day, number of embryos transferred, number of eggs retrieved, number of high-quality embryos, blastocysts, and cleavage-stage embryos, parameters, such as age, endometrial thickness, number of embryos transferred, number of high-quality embryos, and ovulation induction protocol, were correlated with the clinical pregnancy rate. Age was a risk factor for clinical pregnancy rate, whereas endometrial thickness, number of embryos transferred, number of high-quality embryos, and GnRH-a long protocol were protective factors. The older the patient was, the lower her pregnancy rate. The thicker the endometrium, the higher the number of embryos transferred, and the higher the number of high-quality embryos, the higher the pregnancy rate. Compared with the GnRH-ant protocol group, the GnRH-a long protocol group had a higher clinical pregnancy rate (p = 0.001; Table 5).

**Table 5:** Binary logistic regression analysis of factors affecting the clinical pregnancy rate

	OR	95% CI	p value
Age	0.949	0.927–0.972	0.00
Body mass index	1.012	0.982–1.043	0.45
Endometrial thickness on hCG day	1.074	1.009–1.143	0.03
Number of embryos transferred	1.423	1.018–1.990	0.04
Number of eggs retrieved	1.011	0.984–1.039	0.41
Number of high-quality embryos	1.058	1.013–1.106	0.11
Blastocyst	1		
Cleavage-stage embryos	1.013	0.615–1.667	0.96
GnRH-ant protocol	1		
Ultralong GnRH-a protocol	0.838	0.588–1.194	0.33
GnRH-a long protocol	0.709	0.573–0.877	0.02

GnRH-a, gonadotropin-releasing hormone agonist; GnRH-ant, gonadotropin-releasing hormone antagonist; hCG, human chorionic gonadotropin; OR, odds ratio; CI, confidence interval

### Binary logistic regression analysis of factors affecting the early miscarriage rate

After adjusting for factors, such as age, body mass index, endometrial thickness, number of embryos transferred, number of eggs retrieved, number of high-quality embryos, blastocysts, and cleavage-stage embryos, the variable age was correlated with the early miscarriage rate. The older the patient was, the higher the early miscarriage rate. Thus, age was considered a risk factor for early miscarriage (Table 6).

**Table 6:** Binary logistic regression analysis of factors affecting the early miscarriage rate

	OR	95% CI	p-value
Age	1.078	1.031–1.126	0.00
Body mass index	1.048	0.989–1.110	0.11
Endometrial thickness on hCG day	0.975	0.864–1.100	0.68
Number of embryos transferred	0.814	0.442–1.499	0.51
Number of eggs retrieved	1.035	0.983–1.090	0.19
Number of high-quality embryos	1.023	0.943–1.110	0.58
Blastocyst	1		
Cleavage-stage embryos	1.156	0.457–2.920	0.76
GnRH-ant protocol	1		
Ultralong GnRH-a protocol	0.842	0.414–1.714	0.64
GnRH-a long protocol	0.855	0.563–1.299	0.46

GnRH-a, gonadotropin-releasing hormone agonist; GnRH-ant, gonadotropin-releasing hormone antagonist; hCG, human chorionic gonadotropin; OR, odds ratio; CI, confidence interval

## Discussion

IVF-D refers to the procedure of ultrasound-guided egg retrieval after controlled ovarian hyperstimulation in women, followed by fertilization of the retrieved eggs with the thawed donor sperm. The resulting embryos are transferred into the uterine cavity for implantation [7]. Controlled ovarian hyperstimulation refers to the procedure of inducing the simultaneous development and maturation of multiple follicles with drugs to obtain high-quality eggs. The use of individualized controlled ovarian hyperstimulation protocols according to patient characteristics ensures ovulation induction and minimizes the risk of complications. Currently, many studies examine ovulation induction protocols in AID and IVF with partner sperm, but only a few studies have considered IVF-D and whether different ovulation induction protocols affect the associated outcomes. This study aimed to retrospectively analyze the pregnancy outcomes of patients undergoing IVF-D with different ovulation induction protocols in a large sample from Peking University Third Hospital to provide reference data for the selection of appropriate ovulation induction protocols in patients undergoing IVF-D.

The comparison of the three ovulation induction protocols in this study showed that the GnRH-ant protocol had the shortest duration of Gn administration and the lowest Gn dose, compared to the other two protocols. A large amount of Gn may have been released after the binding of GnRH-a to the receptors, and the pituitary function was inhibited after the receptors were stimulated by the agonist in large quantities, which further reduced the production of endogenous Gn. Thus, the endocrine function of the ovary stagnated, reducing the sensitivity of the ovary to exogenous Gn, which increased the administration duration and total dosage of Gn in ovulation induction. In contrast, GnRH-ant blocked the action of endogenous GnRH by binding to the receptors and did not inhibit the pituitary function, thereby shortening the duration of Gn administration and reducing the total Gn dose [8]. The study findings of Wang et al. [9] suggest that, compared with the GnRH-a long protocol, the GnRH-ant protocol reduces the duration and dosage of Gn administration and significantly decreases the incidence of ovarian hyperstimulation syndrome. However, we found no significant differences in either miscarriage or cycle cancelation rates, indicating that the GnRH-ant protocol was more convenient and safer for patients.

The study by Demirdağ et al. [10] showed that the number of eggs retrieved in patients with the GnRH-a protocol was higher than that in patients with the GnRH-ant protocol. According to the results of our study, the GnRH-ant protocol led to the lowest number of eggs retrieved. This is expected considering that the shorter stimulation time and lower Gn dose in the GnRH-ant protocol may lead to reduced oocyte retrieval [9]. The increase in LH during the ovulation induction with the GnRH-ant protocol may lead to the arrest of granulosa cell division, oocyte atresia, premature luteinization, and asynchronous oocyte maturation, thus adversely affecting the development of oocytes [11]. In a cycle with GnRH-ant protocol, the physiological increase in FSH during the luteofollicular transition may lead to uneven follicular development, resulting in a slightly lower production rate of mature oocytes [12].

The results of this study also suggest that the endometrial thickness and clinical pregnancy rate of patients treated with the GnRH-ant protocol were significantly lower than those of the patients treated with the ultralong GnRH-a or GnRH-a long protocol. These results are consistent with the findings of Orvieto et al. [13]. Prolonged downregulation by the GnRH-a protocol may increase the endometrial receptivity in women undergoing IVF treatment, thereby improving the reproductive outcomes [14]. The follicular-phase ultralong GnRH-a protocol can maintain ovarian endocrine function in a state of stagnation, achieving pituitary downregulation, promoting synchronous follicular development during hyperovulation, increasing the number of eggs obtained [13], and improving oocyte and embryo quality by reducing levels of inflammatory factors, such as interleukin-1, interleukin-8, and vascular endothelial growth factor [15]. In patients with normal ovarian function, some studies have shown lower clinical pregnancy rates with antagonist protocols than with agonist protocols [16]. Ruan et al. [17] found that the physiological secretion function of the endometrium was restored, and the uterine receptivity improved, after the application of the GnRH-a protocol. The endometrium expresses GnRH receptors, and GnRH gene expression is present throughout the menstrual cycle, which especially increases in the secretory phase [18]. The GnRH expression in the endometrium may play a paracrine/autocrine role during embryo implantation. Comparative proteomic analysis showed that the GnRH-ant protocol impaired endometrial receptivity significantly more than the GnRH-a protocol [19]. High expression of HOXA10 plays an important role in embryo implantation and endometrial proliferation. Antagonists can negatively affect endometrial receptivity by decreasing the expression of the regulatory protein HOXA10 in the endometrial stroma [20] or by promoting premature maturation of the endometrium [21]. HOXA10 expression in the endometrium is significantly decreased during the GnRH-ant protocol compared to the GnRH-a protocol [22]. In addition, the upregulated expression of allograft inflammatory factor-1 (AIF-1) in the endometrium during the GnRH-ant protocol may be detrimental to embryo implantation. AIF-1, a cytokine associated with inflammation and allograft rejection, may inhibit adhesion during implantation by increasing tumor necrosis factor-α levels [23]. LH stimulates follicular membrane cells to produce androgens, and low-dose LH can upregulate LH receptor content in follicular membrane cells, inducing the formation of LH receptors in granulosa cells. Therefore, follicular development requires a certain level of LH. Some studies have shown that LH instability during GnRH antagonist cycles reduces the probability of pregnancy [24].

It has been reported that for female patients under 30 years old with good ovarian reserve and high risk of ovarian hyperstimulation syndrome, the cumulative pregnancy rate is significantly increased using the GnRH-ant protocol to perform whole-embryo cryopreservation without transfer during the fresh cycle [25]. However, cumulative pregnancy rate statistics were not performed in this study. The absence of cumulative pregnancy rates (from both fresh and frozen cycles) limits the ability to draw conclusions about the overall protocol efficacy.

This study has some limitations. First, in this study, only pregnancy outcomes in fresh cycles were examined. Cycles of whole-embryo cryopreservation with GnRH-ant protocol for ovarian hyperstimulation syndrome prevention were not included, and the pregnancy and cumulative pregnancy rates in thaw cycles with different protocols were not investigated. Future studies should evaluate these protocols using larger datasets. Second, this study was retrospective in design, possibly leading to protocol selection bias due to the different ovarian functions or patient characteristics across the female patient groups. However, we adjusted for covariates in binary regression analyses. Studies with large sample sizes are needed to confirm the present results.

In conclusion, in fresh embryo transfer cycles with three protocols, the GnRH-ant protocol group had the shortest duration of Gn administration with the lowest total Gn dose, whereas the GnRH-a long protocol group had the highest clinical pregnancy rate. Therefore, the GnRH-a long protocol is considered the preferred protocol for female patients who can undergo fresh transfers during IVF-D cycles.

## Data Availability

No datasets were generated or analysed during the current study.

## References

[1] World Health Organization (2023) Infertility prevalence estimates, 1990–2021. World Health Organization. https://www.who.int/publications/i/item/978920068315

[2] Jarow JP, Espeland MA, Lipshultz LI (1989) Evaluation of the azoospermic patient. J Urol 142:62–65. 10.1016/s0022-5347(17)38662-7 2499695 10.1016/s0022-5347(17)38662-7

[3] Porter RN, Smith W, Craft IL, Abdulwahid NA, Jacobs HS (1984) Induction of ovulation for in vitro fertilization using buserelin and gonadotropins. Lancet 2:1284–1285. 10.1016/s0140-6736(84)92840-x 6150318 10.1016/s0140-6736(84)92840-x

[4] Hughes EG, Fedorkow DM, Daya S, Sagle MA, Van de Koppel P, Collins JA (1992) The routine use of gonadotropin-releasing hormone agonists prior to in vitro fertilization and gamete intrafallopian transfer: a meta-analysis of randomized controlled trials. Fertil Steril 58:888–896. 10.1016/s0015-0282(16)55430-2 1426372 10.1016/s0015-0282(16)55430-2

[5] Coccia ME, Comparetto C, Bracco GL, Scarselli G (2004) GnRH antagonists. Eur J Obstet Gynecol Reprod Biol 115(Suppl 1):S44–S56. 10.1016/j.ejogrb.2004.01.033 15196716 10.1016/j.ejogrb.2004.01.033

[6] Hu LL, Huang GN, Sun HX, Fan LQ, Feng Y, Shen H, Sun Y (2018) CSRM consensus on key indicators for quality control in ART clinical operation. J Reprod Med 27:828–835

[7] Xin ZM, Xu B, Jin HX, Song WY, Sun YP (2012) Day 3 embryo transfer may have better pregnancy outcomes in younger than 35-year-old patients with poor ovarian response. J Assist Reprod Genet 29:1077–1081. 10.1007/s10815-012-9830-y 23011285 10.1007/s10815-012-9830-y PMC3492572

[8] Marci R, Caserta D, Lisi F, Graziano A, Soave I, Lo Monte G, Patella A, Moscarini M (2013) In vitro fertilization stimulation protocol for normal responder patients. Gynecol Endocrinol 29:109–112. 10.3109/09513590.2012.712002 22943624 10.3109/09513590.2012.712002

[9] Wang R, Lin S, Wang Y, Qian W, Zhou L (2017) Comparisons of GnRH antagonist protocol versus GnRH agonist long protocol in patients with normal ovarian reserve: a systematic review and meta-analysis. PLoS ONE 12:e0175985. 10.1371/journal.pone.0175985 28437434 10.1371/journal.pone.0175985 PMC5402978

[10] Demirdağ E, Akdulum MFC, Guler I, Oguz Y, Erdem A, Erdem M (2021) IVF outcomes of microdose flare-up, GNRH antagonist, and long protocols in patients having a poor ovarian response in the first treatment cycle. J Coll Physicians Surg Pak 30:523–527. 10.29271/jcpsp.2021.05.523 10.29271/jcpsp.2021.05.523 34027862

[11] Esposito MA, Barnhart KT, Coutifaris C, Pasquale P (2001) Role of periovulatory luteinizing hormone concentrations during assisted reproductive technology cycles stimulated exclusively with recombinant follicle-stimulating hormone. Fertil Steril 75:519–524. 10.1016/s0015-0282(00)01745-3 11239535 10.1016/s0015-0282(00)01745-3

[12] Park CW, Hwang YI, Koo HS, Kang IS, Yang KM, Song IO (2014) Early gonadotropin-releasing hormone antagonist start improves follicular synchronization and pregnancy outcome as compared to the conventional antagonist protocol. Clin Exp Reprod Med 41:158–164. 10.5653/cerm.2014.41.4.158 25599038 10.5653/cerm.2014.41.4.158 PMC4295942

[13] Orvieto R, Meltzer S, Rabinson J, Zohav E, Anteby EY, Nahum R (2008) GnRH agonist versus GnRH antagonist in ovarian stimulation: the role of endometrial receptivity. Fertil Steril 90:1294–1296. 10.1016/j.fertnstert.2007.10.022 18178197 10.1016/j.fertnstert.2007.10.022

[14] Geng Y, Xun Y, Hu S, Lai Q, Jin L (2019) GnRH antagonist versus follicular-phase single-dose GnRH agonist protocol in patients of normal ovarian responses during controlled ovarian stimulation. Gynecol Endocrinol 35:309–313. 10.1080/09513590.2018.1528221 30430883 10.1080/09513590.2018.1528221

[15] Xia M, Zheng J (2021) Comparison of clinical outcomes between the depot gonadotrophin-releasing hormone agonist protocol and gonadotrophin-releasing hormone antagonist protocol in normal ovarian responders. BMC Pregnancy Childbirth 21:372. 10.1186/s12884-021-03849-8 33975553 10.1186/s12884-021-03849-8 PMC8112052

[16] Al-Inany HG, Abou-Setta AM, Aboulghar M (2007) Gonadotrophin-releasing hormone antagonists for assisted conception: a Cochrane review. Reprod Biomed Online 14:640–649. 10.1016/s1472-6483(10)61059-0 17509210 10.1016/s1472-6483(10)61059-0

[17] Ruan HC, Zhu XM, Luo Q, Liu AX, Qian YL, Zhou CY, Jin F, Huang HF, Sheng JZ (2006) Ovarian stimulation with GnRH agonist, but not GnRH antagonist, partially restores the expression of endometrial integrin beta3 and leukemia-inhibitory factor and improves uterine receptivity in mice. Hum Reprod 21:2521–2529. 10.1093/humrep/del215 16790614 10.1093/humrep/del215

[18] Dong KW, Marcelin K, Hsu MI, Chiang CM, Hoffman G, Roberts JL (1998) Expression of gonadotropin-releasing hormone (GnRH) gene in human uterine endometrial tissue. Mol Hum Reprod 4:893–898. 10.1093/molehr/4.9.893 9783851 10.1093/molehr/4.9.893

[19] Chen Q, Yu F, Li Y, Zhang AJ, Zhu XB (2019) Comparative proteomics reveal negative effects of gonadotropin-releasing hormone agonist and antagonist on human endometrium. Drug Des Devel Ther 13:1855–1863. 10.2147/DDDT.S201871 31239640 10.2147/DDDT.S201871 PMC6554521

[20] Rackow BW, Kliman HJ, Taylor HS (2008) GnRH antagonists may affect endometrial receptivity. Fertil Steril 89:1234–1239. 10.1016/j.fertnstert.2007.04.060 18410932 10.1016/j.fertnstert.2007.04.060 PMC2699407

[21] Kolibianakis E, Bourgain C, Albano C, Osmanagaoglu K, Smitz J, Steirteghem AV, Devroey P (2002) Effect of ovarian stimulation with recombinant follicle-stimulating hormone, gonadotropin-releasing hormone antagonists, and human chorionic gonadotropin on endometrial maturation on the day of oocyte pick-up. Fertil Steril 78:1025–1029. 10.1016/s0015-0282(02)03323-x 12413988 10.1016/s0015-0282(02)03323-x

[22] Chen Q, Fan Y, Zhou XW, Yan Z, Kuang YP, Zhang AJ, Xu C (2020) GnRH antagonist alters the migration of endometrial epithelial cells by reducing CKB. Reproduction 159:733–743. 10.1530/REP-19-0578 32213653 10.1530/REP-19-0578

[23] Xu BF, Zhou MJ, Wang JW, Zhang D, Guo F, Si CC, Leung PCK, Zhang A (2018) Increased AIF-1-mediated TNF-α expression during implantation phase in IVF cycles with GnRH antagonist protocol. Hum Reprod 33:1270–1280. 10.1093/humrep/dey119 29897458 10.1093/humrep/dey119 PMC6012176

[24] Seow KM, Lin YH, Hsieh B-C, Huang LW, Huang SC, Chen CY, Chen PH, Tzeng CR, Hwang JL (2010) Characteristics of progesterone changes in women with subtle progesterone rise in recombinant follicle-stimulating hormone and gonadotropin-releasing hormone antagonist cycle. Gynecol Obstet Invest 70:64–68. 10.1159/000290062 20203521 10.1159/000290062

[25] Zhang WL, Xie D, Zhang HD, Huang JL, Xiao XF, Wang BR, Tong Y, Miao Y, Wang X (2020) Cumulative live birth rates after the first art cycle using flexible GnRH antagonist protocol vs. standard long GnRH agonist protocol: a retrospective cohort study in women of different ages and various ovarian reserve. Front Endocrinol (Lausanne) 11:287. 10.3389/fendo.2020.00287 32457698 10.3389/fendo.2020.00287 PMC7225261

